# Supplementary material for: Structures of a FtsZ single protofilament and a double-helical tube in complex with a monobody
Source: Nat Commun. 2023 Jul 10;14:4073. doi: 10.1038/s41467-023-39807-5 (PMC10333351; doi:10.1038/s41467-023-39807-5)
Supplement: Supplementary file 1 — Supplementary Information [file 41467_2023_39807_MOESM1_ESM.pdf]

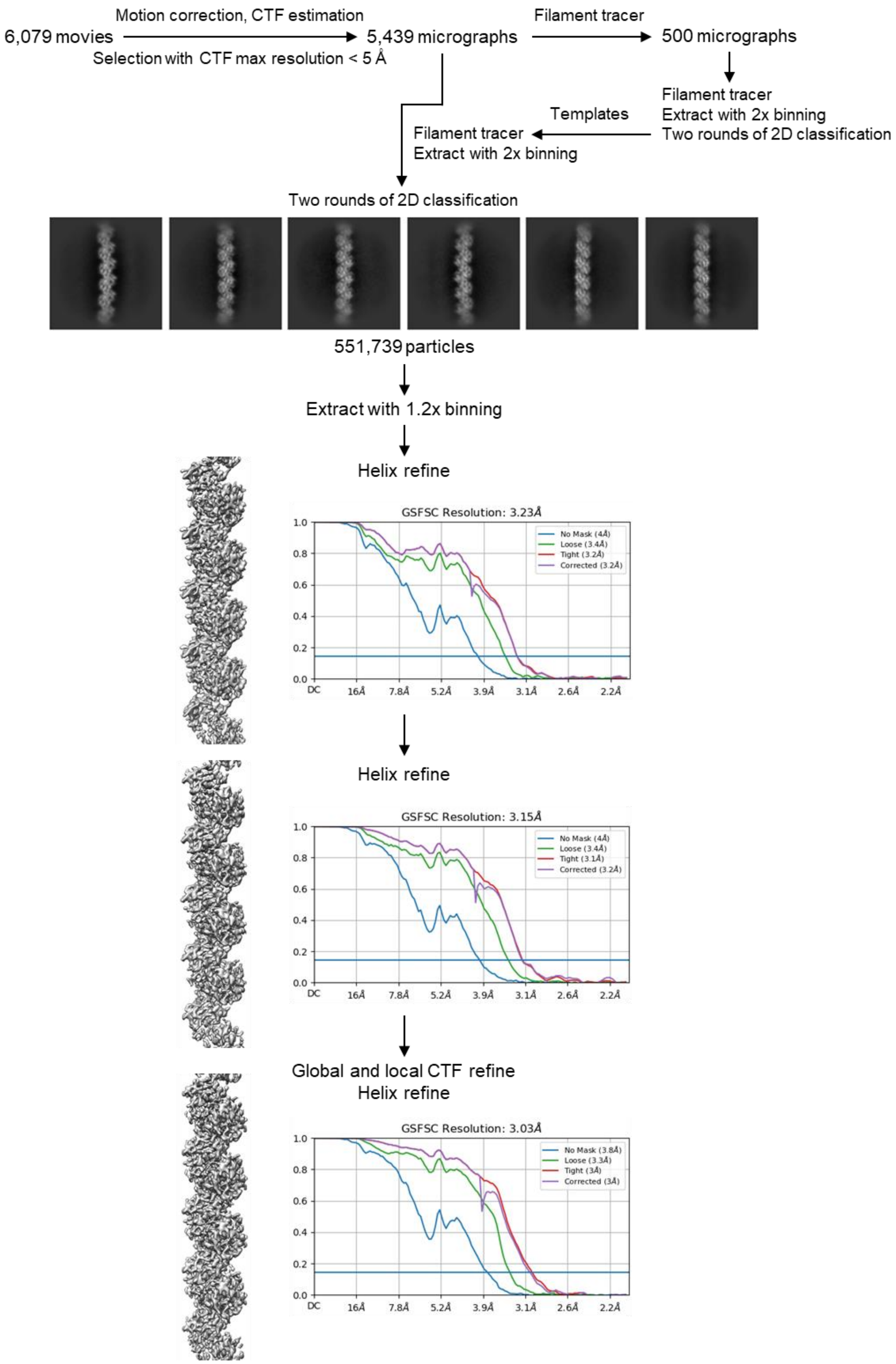

Supplementary Figure 1: CryoEM data processing workflow of KpFtsZ single filament.

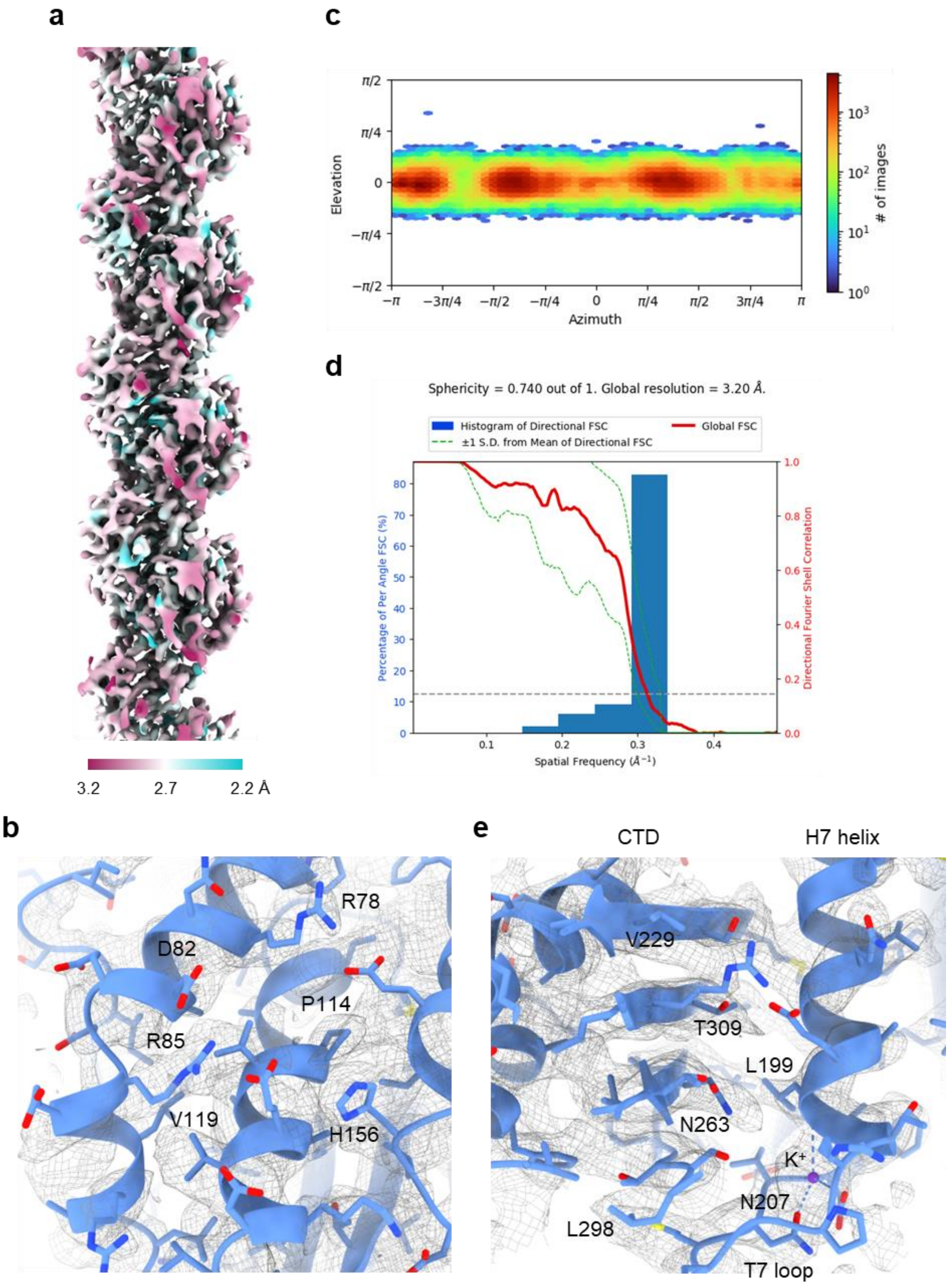

Supplementary Fig. 2: CryoEM analysis of KpFtsZ single filament. a Final sharpened map of KpFtsZ single filament. The local resolution distributions are colored as in the color bar. **b** Close-up view around the N-terminal domain. **c** Angular distribution of the particles used in the final reconstruction. **d** FSC curves and the sphericity calculated by the 3DFSC server (https://3dfsc.salk.edu) for the final map. **e** Close-up view around CTD and the H7 helix. The map contour level is 0.2 for (**b**) and (**e**).

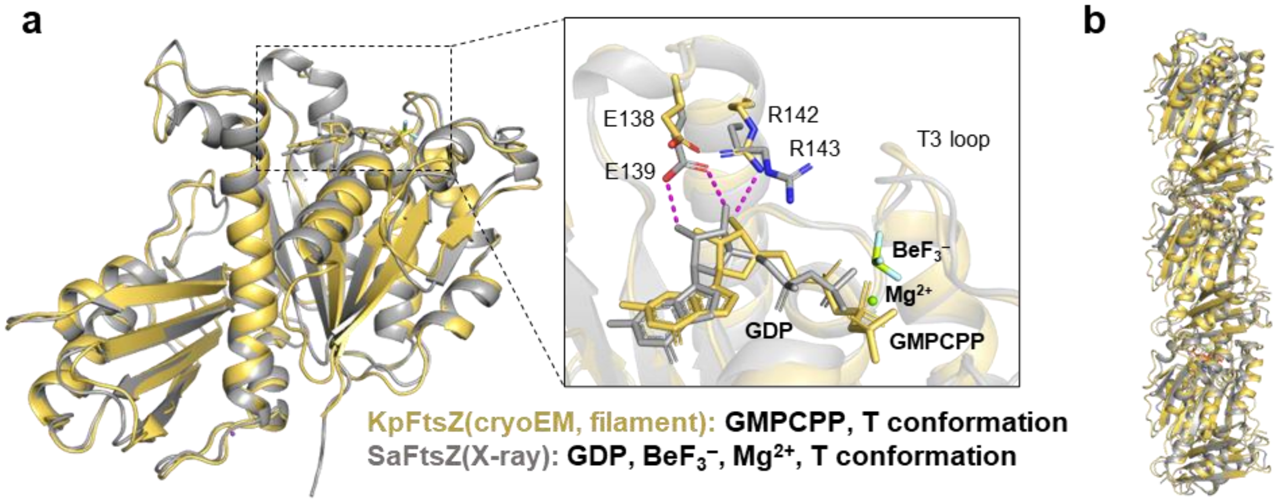

Supplementary Fig. 3: a Superimposition of KpFtsZ in the single protofilament structure and SaFtsZ complexed with GDP, BeF_3^-, and Mg^{2+} in the crystal structure (PDB code:7OHK [http://doi.org/10.2210/pdb7OHK/pdb]). The inset shows a close-up view around GMPCPP/GTPγS. **b** Structural comparison of the FtsZ protofilaments of the two datasets compared in (**a**).

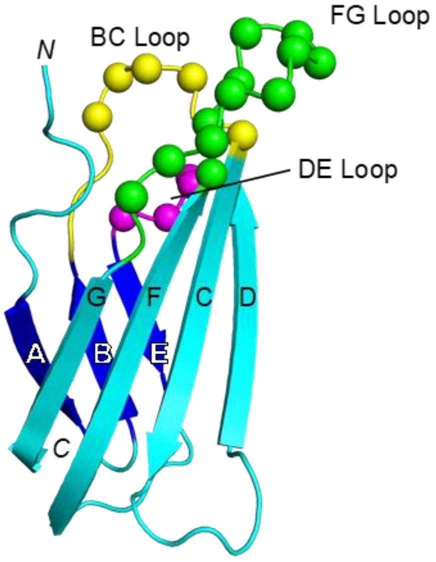

Supplementary Fig. 4: Schematic of the monobody scaffold. The locations of diversified residues in the loop library are shown as spheres, and strands, loops, and termini are labeled.

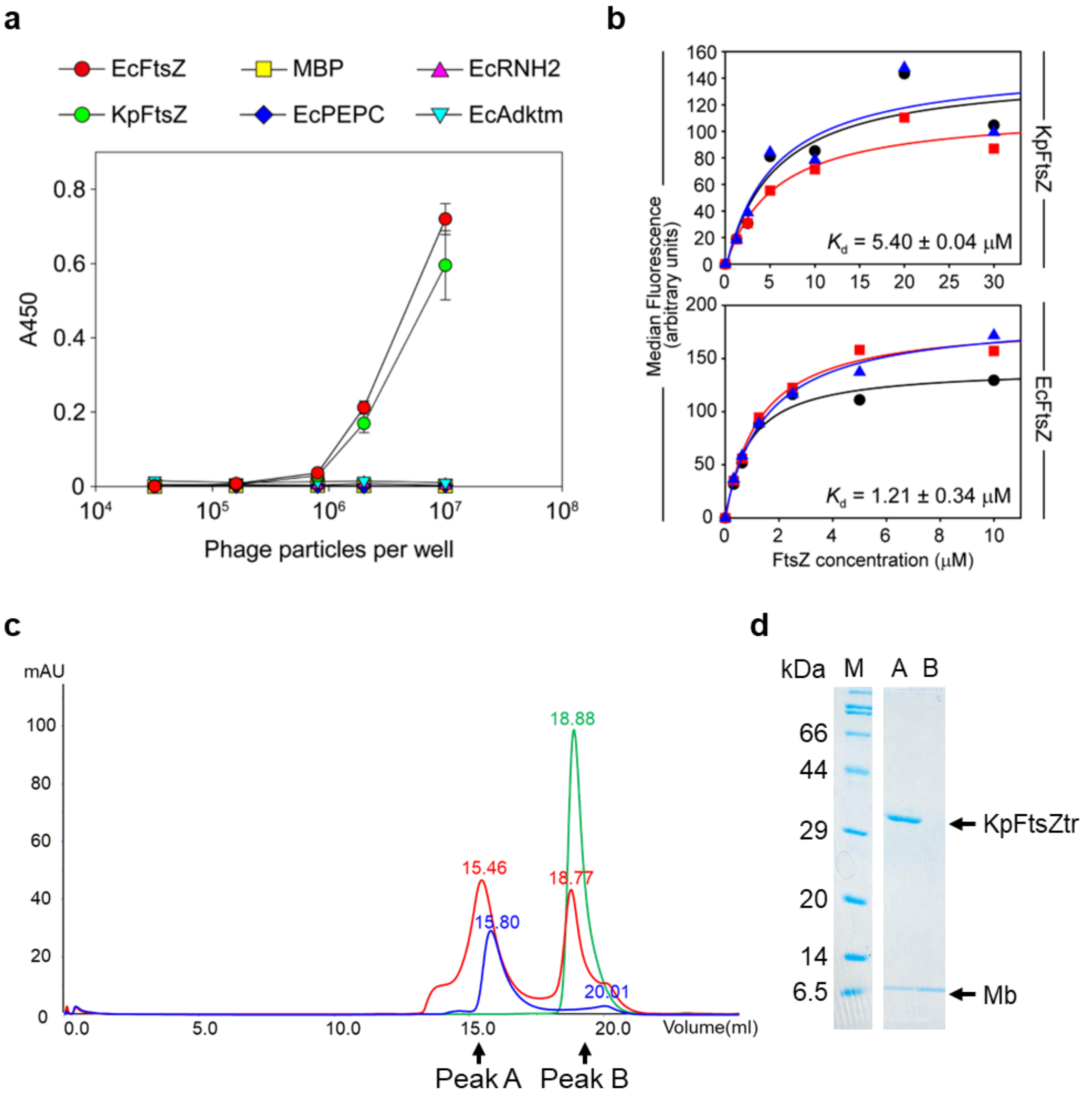

Supplementary Fig. 5: Binding characterization of Mb. **a** Phage ELISA analysis of binding of Mb to its cognate and off-target proteins. The binding of Mb-phages to KpFtsZ- or EcFtsZ-coated wells as a cognate target and MBP-, EcPEPC-, EcRNH2-, or EcAdktm-coated wells as an off-target was measured. ELISA signals are plotted as a function of the number of phages added to a microtiter plate. The data are represented as mean ± s.d. from three independent experiments, and where none are visible, the errors are within the size of the markers. **b** Binding titration curves and the dissociation constants (K_d) of Mb against KpFtsZ (upper panel) and EcFtsZ (lower panel) measured using yeast surface display. The median fluorescence intensities of yeast cells displaying Mb are plotted as a function of the concentration of KpFtsZ or EcFtsZ. The K_d values and errors shown are the mean and standard deviations of three independent measurements. **c** Gel filtration chromatograms of KpFtsZtr (blue curve), Mb (green curve), and the KpFtsZtr–Mb complex (red curve). **d** SDS-PAGE analysis of the different fractions of the KpFtsZtr–Mb complex. ‘M’ denotes protein marker; ‘A’ denotes peak A fraction; ‘B’ denotes peak B fraction. The experiments were repeated twice with similar results for (**c**) and (**d**). Source data are provided as a Source Data file.

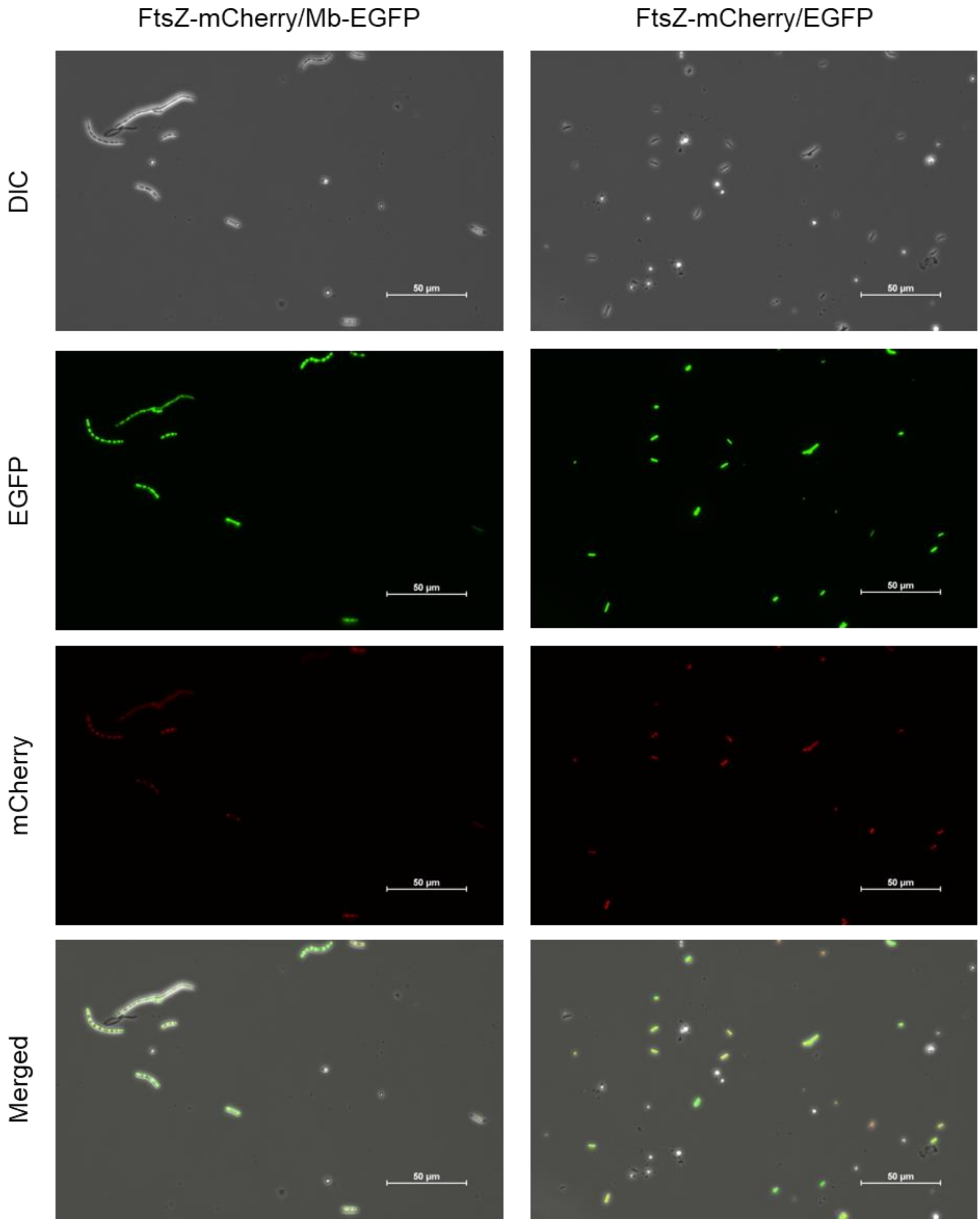

Supplementary Fig. 6: Differential interference contrast (DIC) and fluorescence microscopy images (Scale bar: 50 μm) of *E. coli* cells overproducing EcFtsZ-mCherry/Mb-EGFP and EcFtsZ-mCherry/EGFP. DIC images show that the total lengths of *E. coli* coexpressing FtsZ-mCherry/Mb-EGFP are longer than those of *E. coli* coexpressing FtsZ-mCherry/EGFP. EGFP and mCherry fluorescence images of FtsZ-mCherry/Mb-EGFP show that FtsZ and Mb are localized, respectively. Merged fluorescence images of FtsZ-mCherry/Mb-EGFP confirm that FtsZ and Mb are co-localized in the cells. Each experiment was repeated independently at least three times with similar results.

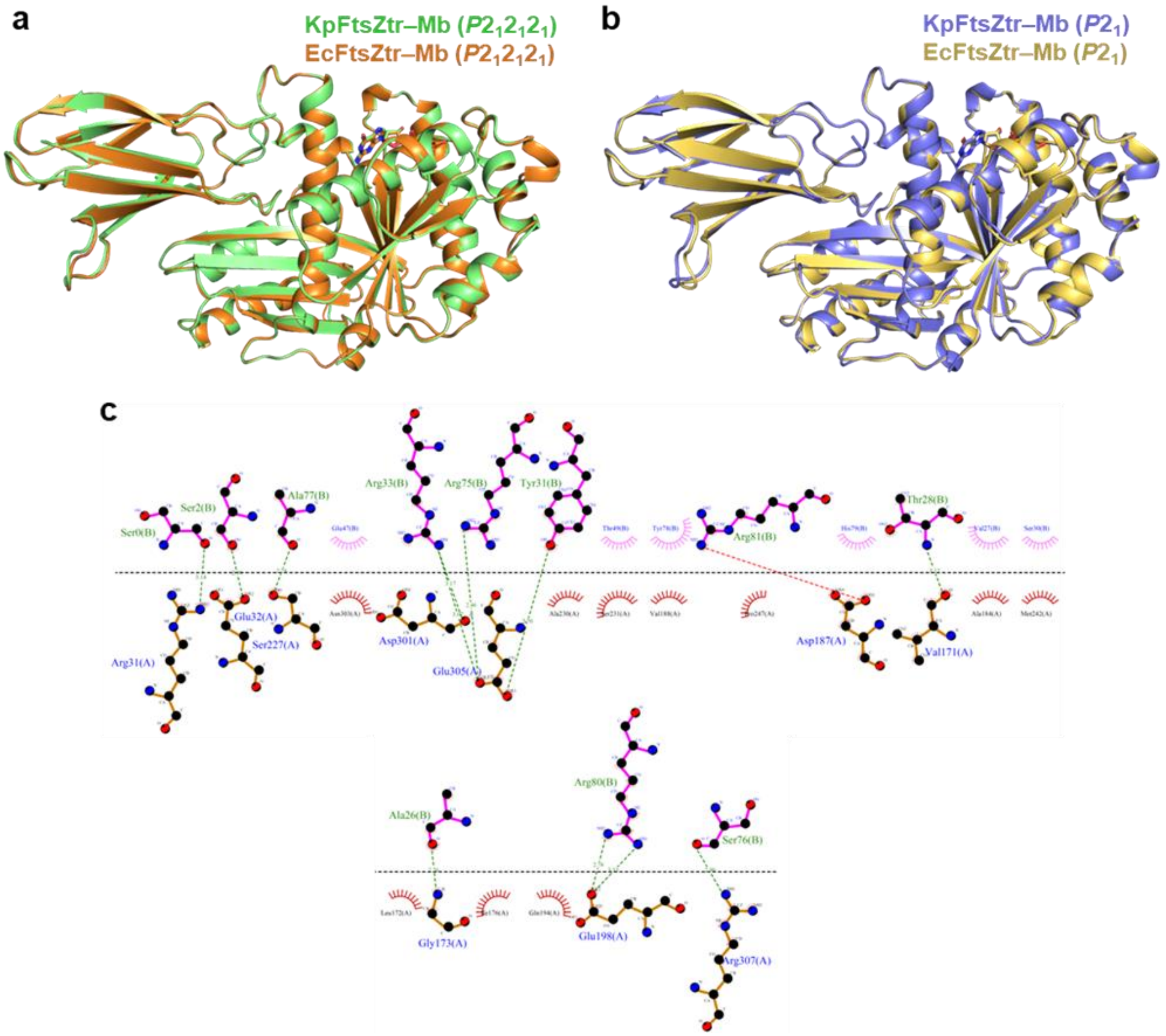

Supplementary Fig. 7: Crystal structures of KpFtsZtr–Mb and EcFtsZtr–Mb complexes. a,b Superimposed crystal structures of the KpFtsZtr–Mb with EcFtsZtr–Mb (**a**) in the $P2_12_12_1$ space group and (**b**) in the $P2_1$ space group. **c** Detailed interfacial interactions between KpFtsZ and Mb in the $P2_12_12_1$ dataset. Interactions were detected and drawn with LigPlot$^+$.

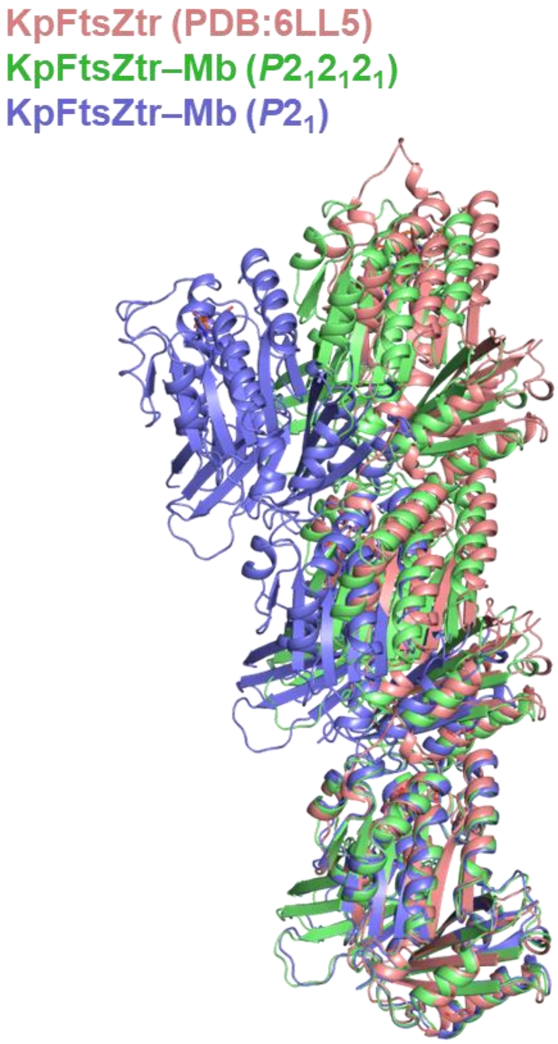

Supplementary Fig. 8: Structure comparison of KpFtsZtr and KpFtsZtr–Mb protofilaments in the two different space groups.

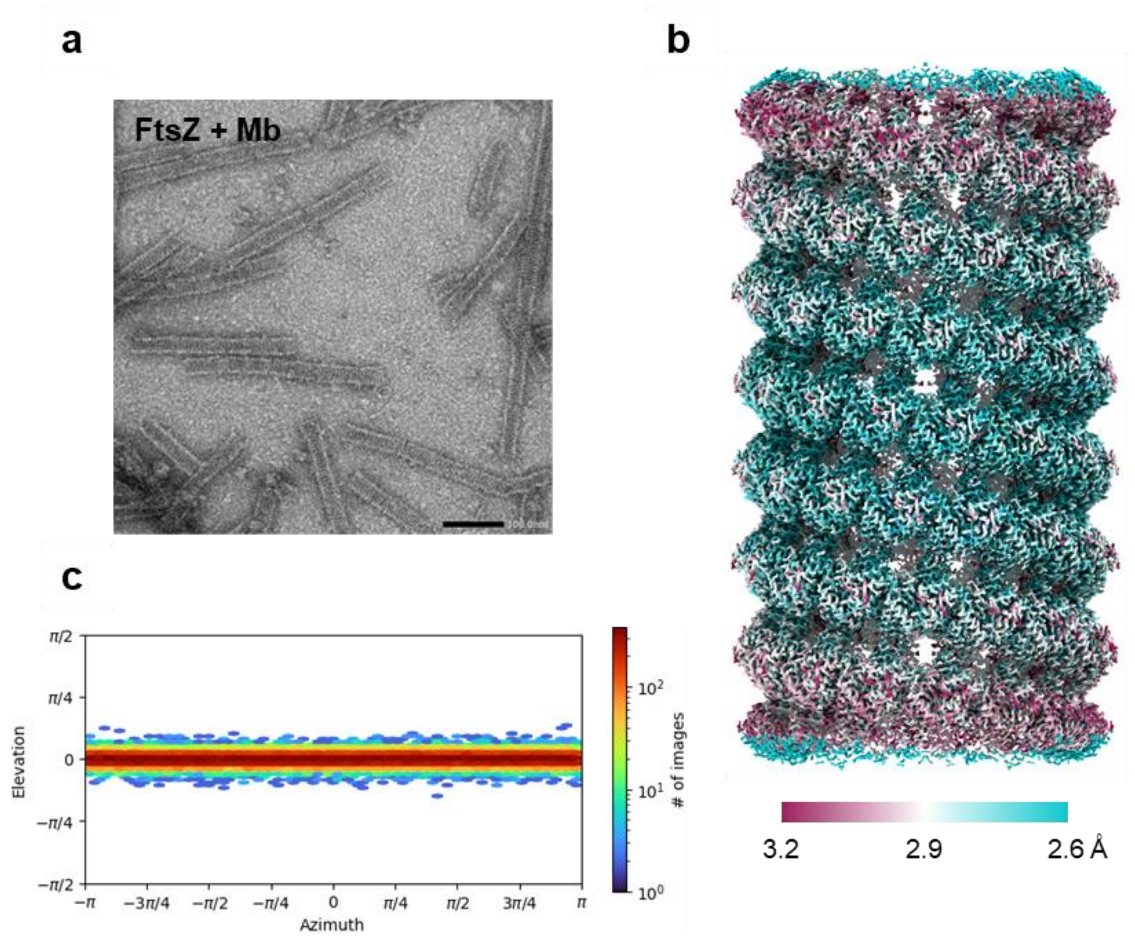

Supplementary Fig. 9: Structure analysis of KpFtsZ–Mb double-helical tube. a Typical negative stain micrograph of 1.0 mg ml^{-1} FtsZ supplemented with 1.2 molar excess of Mb. The scale bar represents 100 nm. The experiment was repeated independently at least twice with similar results. **b** Final sharpened map of FtsZ–Mb double-helical tube. The local resolution distributions are colored as in the color bar. **c** Angular distribution of the particles used in the final reconstruction.

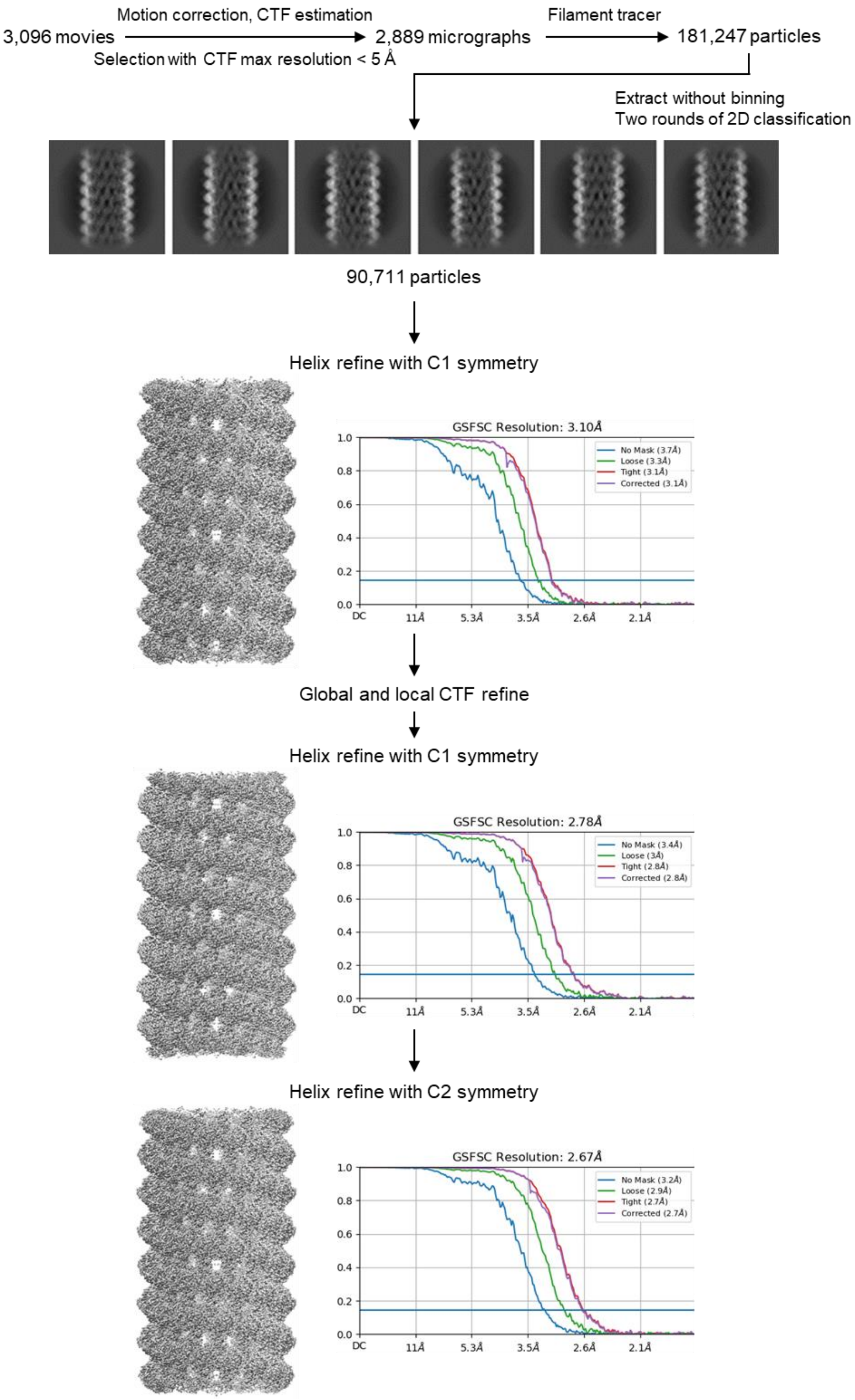

Supplementary Fig. 10: CryoEM data processing workflow of KpFtsZ–Mb double-helical tube.

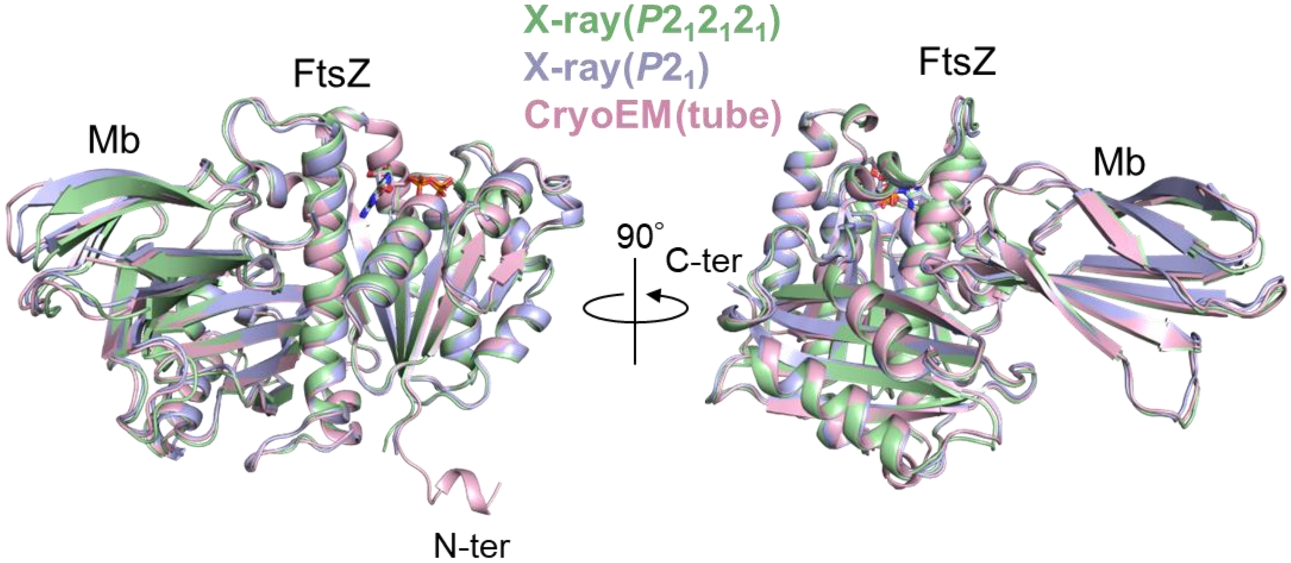

Supplementary Fig. 11: Structure comparison of KpFtsZ monomers in the crystal and CryoEM structures. Two orthogonal views are shown in the left and right panels.

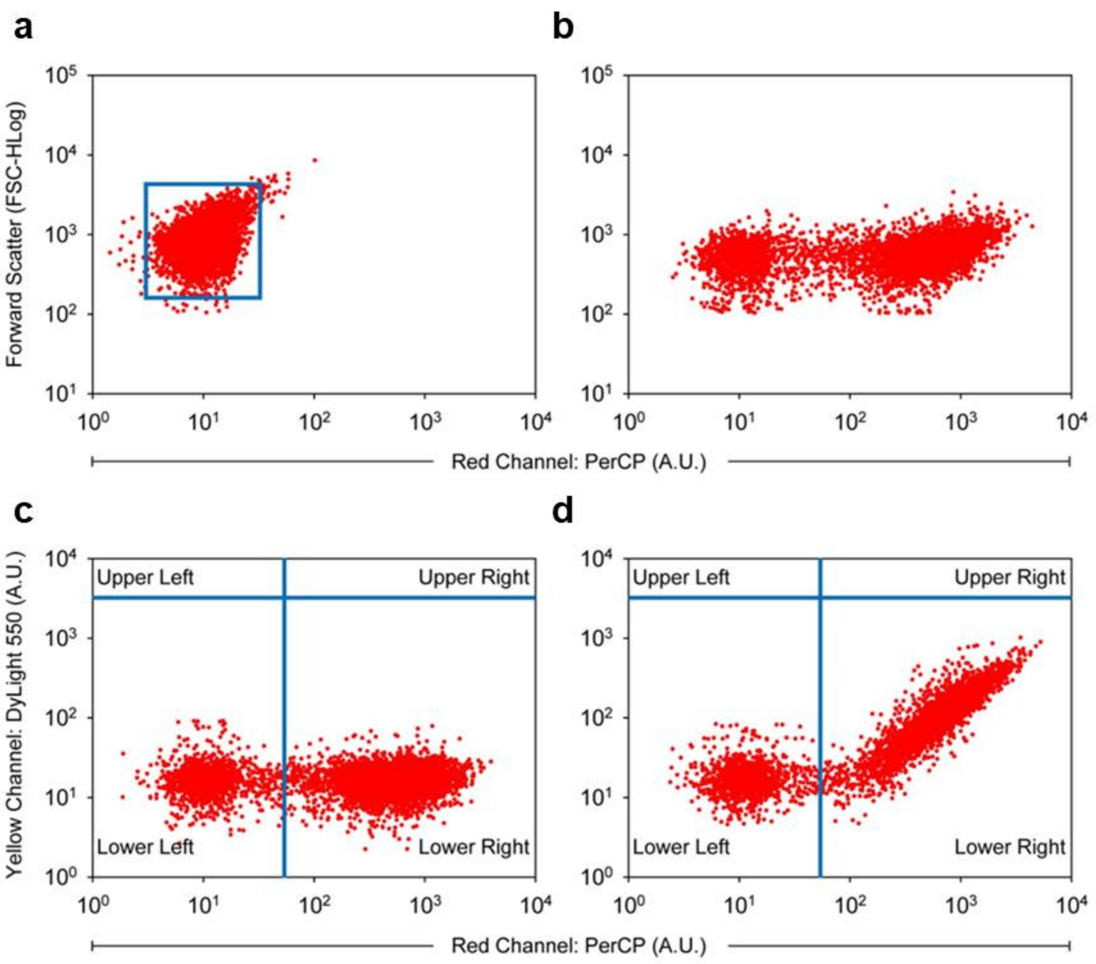

Supplementary Fig. 12: Example of the gating strategy for flow cytometry experiments. a Example of the gating strategy for analyzing yeast cells. The experiments were performed using a single type of non-stained yeast cells, which gave rise to a tight distribution on FSC vs red fluorescence plot. The gate in blue was used for subsequent analysis of the fluorescent population. **b** Example of FSC vs red fluorescence plot for Mb-expressing yeast cells stained with PerCP to assess the monobody expression. **c-d** Example gating strategy for target binding experiments. For these experiments, Mb-expressing yeast cells were incubated in the absence (**c**) or the presence (**d**) of FtsZ, followed by staining with PerCP and DyLight 550 to assess the monobody expression and target binding, respectively. The binding of Mb to FtsZ was measured by taking the median fluorescence intensity (MFI) of 75–90% of the DyLight 550 fluorescent population in the lower right quadrant. For panel (**d**), the target protein was EcFtsZ at the concentration of 1,250 nM.

Supplementary Table 1: CryoEM data collection and processing

	KpFtsZ single filament (EMD-35344) (PDB 8IBN)	KpFtsZ–Mb double-helical tube (EMD-34429) (PDB 8H1O)
**Data collection and processing**		
Magnification	60,000	60,000
Voltage (kV)	300	300
Electron exposure ($e^{-}/Å^2$)	60	60
Defocus range (μm)	−0.5 to −2.0	−0.5 to −2.0
Pixel size (Å)	1.034	0.883
Symmetry imposed	C1	C2 helical
Imported movies (no.)	6,079	3,096
Initial particle images (no.)	3,695,707	181,247
Final particle images (no.)	551,739	90,711
Map resolution (Å)	3.03	2.67
FSC threshold	0.143	0.143
Helical parameters		
Rise (Å)	44.02	7.70
Twist (°)	0.025	−23.40
**Refinement**		
Initial model used (PDB code)	6LL5	8GZV
Resolution estimates (Å)		
d model	3.5	2.9
d FSC model (0/0.143/0.5)	3.0/3.1/7.2	2.6/2.6/2.8
Model vs. Data CC (mask)	0.53	0.87
(volume)	0.52	0.82
Model composition		
Non-hydrogen atoms	8944	2975
Protein residues	1224	404
Ligands	8 (GMPCPP and K)	1 (GDP)
R.m.s. deviations		
Bond lengths (Å)	0.004	0.003
Bond angles (°)	0.854	0.532
Validation		
MolProbity score	1.72	1.27
Clashscore	8.05	5.05
Poor rotamers (%)	0	0
Ramachandran plot		
Favored (%)	96.0	98.8
Allowed (%)	4.0	1.2
Disallowed (%)	0	0

Supplementary Table 2: Crystallographic data collection statistics of KpFtsZtr–Mb and EcFtsZtr–Mb complexes

	KpFtsZtr–Mb ($P2_12_12_1$) (PDB 8GZV)	KpFtsZtr–Mb ($P2_1$) (PDB 8GZW)	EcFtsZtr–Mb ($P2_12_12_1$) (PDB 8GZX)	KpFtsZtr–Mb ($P2_1$) (PDB 8GZY)
Space group	$P2_12_12_1$	$P2_1$	$P2_12_12_1$	$P2_1$
Cell dimensions				
a, b, c (Å)	45.29, 64.56, 124.65	88.47, 66.93, 102.17	45.11, 63.30, 123.05	89.34, 66.23, 102.79
α, β, γ (°)	90.00, 90.00, 90.00	90.00, 92.06, 90.00	90.00, 90.00, 90.00	90.00, 92.67, 90.00
Total reflections	250,724 (23,545)*	287,239 (30,389)	412,374 (39,436)	253,395 (26,839)
Unique reflections	19,223 (1,877)	41,534 (4,148)	31,362 (3,066)	37,170 (3,686)
Resolution range	35.54–2.20 (2.28–2.20)	40.05–2.50 (2.59–2.50)	35.2–1.84 (1.91–1.84)	35.25–2.60 (2.69–2.60)
R_{merge}	0.09836 (0.9052)	0.07902 (0.7059)	0.09167 (1.621)	0.05329 (0.6137)
$I/\sigma I$	17.67 (4.06)	15.57 (2.62)	19.50 (1.77)	19.95 (2.96)
Completeness (%)	99.86 (99.73)	99.69 (99.86)	99.95 (100.0)	99.6 (99.8)
Redundancy	13.0 (12.5)	6.9 (7.3)	13.1 (12.9)	6.8 (7.3)
$CC_{1/2}$	0.999 (0.938)	0.998 (0.866)	0.999 (0.661)	0.999 (0.925)
**Refinement**				
R_{work}/R_{free}	0.205/0.264	0.215/0.284	0.205/0.243	0.214/0.296
No. of atoms				
Protein	2,897	8,684	2,870	8,607
Ligand	28	84	28	84
Water	46	40	123	9
B factors (Å^2)				
Protein	46.13	57.15	35.17	83.87
Ligand	53.20	58.82	34.91	76.91
Water	40.70	43.83	36.52	76.24
R. m. s. deviations				
Bond length (Å)	0.008	0.008	0.007	0.009
Bond angles (°)	0.99	1.08	1.05	1.12

*Each dataset was collected from one crystal. *Values in parentheses are for highest-resolution shell.

Supplementary Table 3: Interface areas between two KpFtsZ molecules in a protofilament

Dataset	Pair of molecules	Interface area (Å^2)	ΔG (kcal/mol)
X-ray ($P2_12_12_1$)	-	703.3	–9.7
X-ray ($P2_1$)	A–B	950.5	–14.9
	B–C	962.0	–13.3
CryoEM (double-helical tube)	-	1066.5 (mean)	–14.0 (mean)
CryoEM (single filament)	-	1113.6 (mean)	–17.8 (mean)

Supplementary Table 4: List of synthesized genes used in this study and their sequences

Name	Sequence
KpFtsZ_full	5′-CCAGGGTCATATGTTCGAGCCTATGGAACTCACCAACGACGCGGTTATCAAAGTT<br>ATCGGTGTTGGTGGCGGCGGTGGCAATGCGGTTGAACACATGGTTCGTGAACGTATCGA<br>AGGTGTTGAGTTCTTCGCAGTCAATACCGACGCGCAGGCCCTGCGTAAAACTGCGGTAG<br>GTCAGACGATCCAGATCGGCTCTGGCATCACCAAAGGTCTGGGTGCGGGTGCGAACCCG<br>GAAGTTGGTCGTAATGCCGCGGACGAAGACCGCGAAGCGCTCCGTGCGGCGCTGGACGG<br>TGCGGACATGGTATTCATTGCGGCTGGCATGGGTGGTGGTACGGGTACTGGCGCTGCTC<br>CGGTAGTTGCGGAAGTGGCGAAAGACCTCGGTATCCTGACCGTCGCGGTTGTTACTAAG<br>CCGTTCAACTTCGAGGGCAAAAAACGTATGGCGTTCGCGGAACAGGGTATCACTGAACT<br>CTCTAAACACGTTGACTCTCTGATTACCATCCCGAATGACAAACTGCTGAAAGTGCTGG<br>GTCGTGGCATCTCTCTGCTCGATGCCTTCGGTGCAGCGAACGACGTCCTGAAAGGTGCG<br>GTTCAAGGCATTGCAGAACTGATCACCCGTCCTGGTCTGATGAACGTGGACTTCGCTGA<br>CGTTCGTACCGTTATGTCTGAAATGGGTTACGCGATGATGGGTTCTGGCGTTGCGTCTG<br>GCGAGGATCGCGCCGAAGAGGCGGCAGAAATGGCGATCTCTTCCCCGCTGCTGGAAGAC<br>ATCGACCTGTCTGGTGCGCGTGGTGTTCTGGTTAACATCACCGCGGGTTTTGACCTGCG<br>TCTCGACGAGTTCGAAACCGTTGGTAACACCATTCGTGCATTCGCGTCCGACAACGCGA<br>CCGTAGTTATTGGCACCTCTCTGGACCCGGATATGAATGACGAGCTGCGTGTTACTGTT<br>GTTGCGACGGGTATCGGTATGGACAAACGTCCGGAAATCACCCTGGTTACGAATAAACA<br>GGTTCAGCAACCGGTCATGGACCGCTACCAACAGCACGGTATGTCTCCGCTGACCCAGG<br>AACAGAAACCTGCCGCGAAAGTTGTTAACGACAATACCCCGCAGACCGCAAAAGAACCG<br>GACTACCTGGATATTCCGGCGTTTCTGCGCAAACAGGCGGACTAAGGATCCGAATTCAA<br>GC-3′
EcFtsZ_235-1,107	5′-AATGCCGCGGACGAAGACCGCGATGCGCTCCGTGCGGCGCTGGAAGGTGCGGACA<br>TGGTATTCATTGCGGCTGGCATGGGTGGTGGTACGGGTACTGGCGCTGCTCCGGTAGTT<br>GCGGAAGTGGCGAAAGACCTCGGTATCCTGACCGTCGCGGTTGTTACTAAGCCGTTCAA<br>CTTCGAGGGCAAAAAACGTATGGCGTTCGCGGAACAGGGTATCACTGAACTCTCTAAAC<br>ACGTTGACTCTCTGATTACCATCCCGAATGACAAACTGCTGAAAGTGCTGGGTCGTGGC<br>ATCTCTCTGCTCGATGCCTTCGGTGCAGCGAACGACGTCCTGAAAGGTGCGGTTCAAGG<br>CATTGCAGAACTGATCACCCGTCCTGGTCTGATGAACGTGGACTTCGCTGACGTTCGTA<br>CCGTTATGTCTGAAATGGGTTACGCGATGATGGGTTCTGGCGTTGCGTCTGGCGAGGAT<br>CGCGCCGAAGAGGCGGCAGAAATGGCGATCTCTTCCCCGCTGCTGGAAGACATCGACCT<br>GTCTGGTGCGCGTGGTGTTCTGGTTAACATCACCGCGGGTTTTGACCTGCGTCTCGACG<br>AGTTCGAAACCGTTGGTAACACCATTCGTGCATTCGCGTCCGACAACGCGACCGTAGTT<br>ATTGGCACCTCTCTGGACCCGGATATGAATGACGAGCTGCGTGTTACTGTTGTTGCGAC<br>GGGTATCGGTATGGACAAACGTCCGGAAATCACCCTGGTTACGAATAAACAGGTTCAGC<br>AACCGGTCATGGACCGCTACCAACAGCACGGTATGTATCCGCTGACCCAGGAACAGAAA<br>CCTGTCGCGAAAGTTGTTAACGACAATGCCCCGCAGACCGCAAAAGAACCG-3′

Supplementary Table 5: List of primers used in this study and their sequences

Name	Sequence
pCold-F	5′-ACGCCATATCGCCGAAAGG-3′
pCold-R	5′-GGCAGGGATCTTAGATTCTG-3′
T7P	5′-TAATACGACTCACTATAGGG-3′
T7T	5′-GCTAGTTATTGCTCAGCGG-3′
FN5’-1	5′-GATCCGTTTCTTCTGTTCCGACCAAACTGGAA-3′
FN5’-2	5′-CGTTTCTTCTGTTCCGACCAAACTGGAA-3′
FN3’-1	5′-GTTACTAGGTACGGTAGTTAATCGAGATTGG-3′
FN3’-2	5′-TCGAGTTACTAGGTACGGTAGTTAATCGAGATTGG-3′
MBP-Fw-BamHI	5′-ATTATAGGATCCAAAATCGAAGAAGGTAAACTGGTAATCTGGATTAA-3′
MBP-Rv-XhoI	5′-ATTATACTCGAGTTACGAGCTCGAATTAGTCTGCGCGTCTTTCAGGGCTT-3′
EcPEPC-Fw-BamHI	5′-ATTATAGGATCCATGAACGAACAATATTCCGCATTGCGTAGT
EcPEPC-Rv-XhoI	5′-ATTATACTCGAGTTAGCCGGTATTACGCATACCTGCCGCAAT
EcRNH2-Fw-BamHI	5′-ATTATAGGATCCATCGAATTTGTTTATCCGCATACGCAGCTG
EcRNH2-Rv-XhoI	5′-ATTATACTCGAGGGACGCAAGTCCCAGTGCGCGTTTGACAGG
EcAdktm-Fw-BamHI	5′-ATTATAGGATCCATGCGTATCATTCTGCTTGGCGCTCCGGGC
EcAdktm-Rv-XhoI	5′-ATTATACTCGAGTTACTAGCCGAGGATTTTTTCCAGATCAGCGCGAAC

Supplementary Table 6: Nucleotide sequences of the coding regions of fluorescent protein-fused constructs

Name	Sequence
EcFtsZfull-mCherry	5′-ATGAATCACAAAGTGCATCATCATCATCATCATCTGCAGGAGAATCTTTATTTCCA<br>GGGTCATATGTTCGAGCCTATGGAACTCACCAACGACGCGGTTATCAAAGTTATCGGTG<br>TTGGTGGCGGCGGTGGCAATGCGGTTGAACACATGGTTCGTGAACGTATCGAAGGTGTT<br>GAGTTCTTCGCAGTCAATACCGACGCGCAGGCCCTGCGTAAAACTGCGGTAGGTCAGAC<br>GATCCAGATCGGCTCTGGCATCACCAAAGGTCTGGGTGCGGGTGCGAACCCGGAAGTTG<br>GTCGTAATGCCGCGGACGAAGACCGCGATGCGCTCCGTGCGGCGCTGGAAGGTGCGGAC<br>ATGGTATTCATTGCGGCTGGCATGGGTGGTGGTACGGGTACTGGCGCTGCTCCGGTAGT<br>TGCGGAAGTGGCGAAAGACCTCGGTATCCTGACCGTCGCGGTTGTTACTAAGCCGTTCA<br>ACTTCGAGGGCAAAAAACGTATGGCGTTCGCGGAACAGGGTATCACTGAACTCTCTAAA<br>CACGTTGACTCTCTGATTACCATCCCGAATGACAAACTGCTGAAAGTGCTGGGTCGTGG<br>CATCTCTCTGCTCGATGCCTTCGGTGCAGCGAACGACGTCCTGAAAGGTGCGGTTCAAG<br>GCATTGCAGAACTGATCACCCGTCCTGGTCTGATGAACGTGGACTTCGCTGACGTTCGT<br>ACCGTTATGTCTGAAATGGGTTACGCGATGATGGGTTCTGGCGTTGCGTCTGGCGAGGA<br>TCGCGCCGAAGAGGCGGCAGAAATGGCGATCTCTTCCCCGCTGCTGGAAGACATCGACC<br>TGTCTGGTGCGCGTGGTGTTCTGGTTAACATCACCGCGGGTTTTGACCTGCGTCTCGAC<br>GAGTTCGAAACCGTTGGTAACACCATTCGTGCATTCGCGTCCGACAACGCGACCGTAGT<br>TATTGGCACCTCTCTGGACCCGGATATGAATGACGAGCTGCGTGTTACTGTTGTTGCGA<br>CGGGTATCGGTATGGACAAACGTCCGGAAATCACCCTGGTTACGAATAAACAGGTTCAG<br>CAACCGGTCATGGACCGCTACCAACAGCACGGTATGTATCCGCTGACCCAGGAACAGAA<br>ACCTGTCGCGAAAGTTGTTAACGACAATGCCCCGCAGACCGCAAAAGAACCGGACTACC<br>TGGATATTCCGGCGTTTCTGCGCAAACAGGCGGACGGTGGATCTGGTGGATCAGGCATG<br>GTAAGCAAAGGTGAAGAGGACAACATGGCGATTATCAAAGAGTTTATGCGCTTTAAAGT<br>GCACATGGAAGGTAGCGTTAATGGTCATGAGTTCGAAATTGAAGGTGAAGGCGAAGGCC<br>GTCCGTATGAAGGCACCCAGACTGCGAAACTGAAGGTCACAAAGGGCGGCCCTCTCCCG<br>TTCGCCTGGGACATTCTGAGCCCGCAGTTTATGTATGGCTCCAAAGCCTACGTCAAACA<br>TCCAGCCGATATCCCCGACTATCTGAAACTGAGCTTTCCGGAGGGTTTCAAATGGGAAC<br>GCGTGATGAACTTCGAAGATGGTGGCGTTGTCACCGTGACACAGGATTCGTCGTTGCAA<br>GATGGCGAATTTATCTACAAAGTGAAACTCCGTGGGACCAATTTTCCGTCGGATGGGCC<br>CGTTATGCAGAAGAAAACCATGGGCTGGGAAGCGTCCTCAGAACGCATGTATCCGGAGG<br>ACGGGGCGTTAAAAGGCGAGATCAAACAGCGGTTAAAGCTGAAAGATGGCGGCCACTAT<br>GATGCAGAAGTGAAAACGACGTACAAAGCGAAGAAGCCGGTTCAACTTCCTGGTGCGTA<br>TAACGTGAACATCAAACTGGATATTACCTCCCACAATGAGGACTACACGATTGTAGAAC<br>AGTATGAGCGTGCGGAAGGTCGCCATTCGACTGGTGGGATGGATGAACTGTACAAATAA<br>-3′
Mb-EGFP	5′-ATGAAACACCACCACCACCATCATCATCATCATCACAGCAGCGACTACAAAGACGA<br>CGATGACAAAGGTGAAAACCTGTACTTCCAGGGATCCGTTTCTTCTGTTCCGACCAAAC<br>TGGAAGTTGTTGCTGCGACCCCGACTAGCCTGCTGATCAGCTGGGATGCTCCTGCCGTT<br>ACCGTGTCTTATTACCGTATCACGTACGGTGAAACCGGTGGTAACTCCCCGGTTCAGGA

ATTCACTGTACCTGGTTCCAAATCTACTGCTACCATCAGCGGCCTGAGCCCGGGTGTCG
ACTATACCATCACTGTATACGCTCGCAGCGCCTACCACCGCCGCTCTCCAATCTCGATT
AACTACCGTACCGGAGGCTCTGGCGGATCAAGCAAGGGCGAGGAGCTGTTCACCGGGGT
GGTGCCCATCCTGGTCGAGCTGGACGGCGACGTAAACGGCCACAAGTTCAGCGTGTCCG
GCGAGGGCGAGGGCGATGCCACCTACGGCAAGCTGACCCTGAAGTTCATCTGCACCACC
GGCAAGCTGCCCGTGCCCTGGCCCACCCTCGTGACCACCCTGACCTACGGCGTGCAGTG
CTTCAGCCGCTACCCCGACCACATGAAGCAGCACGACTTCTTCAAGTCCGCCATGCCCG
AAGGCTACGTCCAGGAGCGCACCATCTTCTTCAAGGACGACGGCAACTACAAGACCCGC
GCCGAGGTGAAGTTCGAGGGCGACACCCTGGTGAACCGCATCGAGCTGAAGGGCATCGA
CTTCAAGGAGGACGGCAACATCCTGGGGCACAAGCTGGAGTACAACTACAACAGCCACA
ACGTCTATATCATGGCCGACAAGCAGAAGAACGGCATCAAGGTGAACTTCAAGATCCGC
CACAACATCGAGGACGGCAGCGTGCAGCTCGCCGACCACTACCAGCAGAACACCCCCAT
CGGCGACGGCCCCGTGCTGCTGCCCGACAACCACTACCTGAGCACCCAGTCCGCCCTGA
GCAAAGACCCCAACGAGAAGCGCGATCACATGGTCCTGCTGGAGTTCGTGACCGCCGCC
GGGATCACTCTCGGCATGGACGAGCTGTACAAGTAG-3′
