# Supplementary material for: Structures of a FtsZ single protofilament and a double-helical tube in complex with a monobody
Source: Nat Commun. 2023 Jul 10;14:4073. doi: 10.1038/s41467-023-39807-5 (PMC10333351; doi:10.1038/s41467-023-39807-5)
Supplement: Supplementary file 3 — Description of Additional Supplementary Files [file 41467_2023_39807_MOESM3_ESM.pdf]

Description of Additional Supplementary Files

File Name: Supplementary Movie 1

Description: CryoEM map and model of KpFtsZ–Mb double helical tube.
